# Self-Assembled Monolayers: Versatile Uses in Electronic Devices from Gate Dielectrics, Dopants, and Biosensing Linkers

**DOI:** 10.3390/mi12050565

**Published:** 2021-05-17

**Authors:** Seongjae Kim, Hocheon Yoo

**Affiliations:** Department of Electronic Engineering, Gachon University, Seongnam 13120, Korea; lml456@gachon.ac.kr

**Keywords:** self-assembled monolayers (SAMs), organic materials, gate dielectrics, SAMFETs, doping, biosensors

## Abstract

Self-assembled monolayers (SAMs), molecular structures consisting of assemblies formed in an ordered monolayer domain, are revisited to introduce their various functions in electronic devices. SAMs have been used as ultrathin gate dielectric layers in low-voltage transistors owing to their molecularly thin nature. In addition to the contribution of SAMs as gate dielectric layers, SAMs contribute to the transistor as a semiconducting active layer. Beyond the transistor components, SAMs have recently been applied in other electronic applications, including as remote doping materials and molecular linkers to anchor target biomarkers. This review comprehensively covers SAM-based electronic devices, focusing on the various applications that utilize the physical and chemical properties of SAMs.

## 1. Introduction

Self-assembled monolayers (SAMs) have been used extensively because of their great advantages, which include molecular assembly, spontaneous formation, and formation by immersion of the substrate in a solution with the precursor molecules [1,2,3]. SAMs are composed of a head group that binds to the surface, an end group that determines the surface’s characteristics after SAM treatment, and a spacer group that connects the head and end groups. The head group includes silane, phosphonic acid, and thiol, and the end group includes amine, methyl, and thiol. The spacer group mainly consists of alkyl carbon chains. Through molecular assembly, SAMs form an extremely strong molecule–substrate interaction, whereby chemical bonds are clearly formed at the interface. Owing to these powerful chemical interactions, SAMs can be used in several applications, especially in electronics that require an ordered domain of chemical components. Electronic devices have been developed comprehensively. To functionalize electronic devices, surface treatments are used to alter the interface between the layers to produce hydrophobic, hydrophilic, or specific molecular surfaces. This review provides an overview of the key contributions of SAMs to electronic devices taking into consideration three main aspects: (1) dielectrics, dielectric interfaces, or the semiconductor itself; (2) dopants to control the device’s properties; and (3) linkers to anchor analytics for biosensors.

## 2. SAMs as Basic Elements of the Device

In the first section, we comprehensively recapitulate the important advances of SAMs in organic thin-film transistors (OTFTs) [4,5,6,7,8]. Since 1990, OTFTs have emerged as soft electronic devices with functional properties that include mechanical flexibility [9,10,11,12,13] and a solution-processed, facile manufacturing process [14,15]. However, the organic semiconductors in OTFTs are vulnerable to charge traps [16,17,18] and leakage current, which frequently occur in oxide dielectrics (i.e., silicon dioxide (SiO_2_) [19,20,21], aluminum oxide (Al_2_O_3_) [22,23], and hafnium oxide (HfO_2_) [24]). Owing to the strong susceptibility of organic semiconductors to charge traps, OTFTs suffer from instability, which causes current–voltage sweep hysteresis [25,26,27], shifts in the threshold voltage (V_TH_) [28,29], and bias stress effects [30,31,32,33]. To address these issues, the treatment of the hydroxylated surfaces of dielectrics is crucial. SAMs exhibit appropriate properties to resolve these hydroxyl dielectric–organic semiconductor interface limitations. As SAMs have a Janus structure, a head group on one side and a tail group on the other [34], reactions with SAMs enable a dramatic change in the properties of a film surface. For example, a silane-based SAM contains a Si-O-H head group that can react with a hydroxyl group on the substrate, forming a covalent R-Si-O-substrate bond [35,36]. Through this mechanism, the dielectric surface can be altered to one that is desirable for the stable and robust operation of OTFTs. Furthermore, SAMs act as dielectrics or semiconductors in OTFTs.

### 2.1. SAM Treatment on SiO_2_ Gate Dielectrics

SiO_2_ has a large energy bandgap of ~9 eV and is used as a gate dielectric in most electronic devices owing to its excellent insulating properties. However, the many hydroxyl groups present on the surface of SiO_2_ can act as traps at the interface with the semiconductor layer. Therefore, if the gate dielectric SiO_2_ film is not controlled, the electrical hysteresis caused by the trap can lead to problems, such as operation failures of the electronic device, which must be solved. Based on this, many researchers have studied the use of SAMs as a surface treatment for SiO_2_.

**Figure 1:**
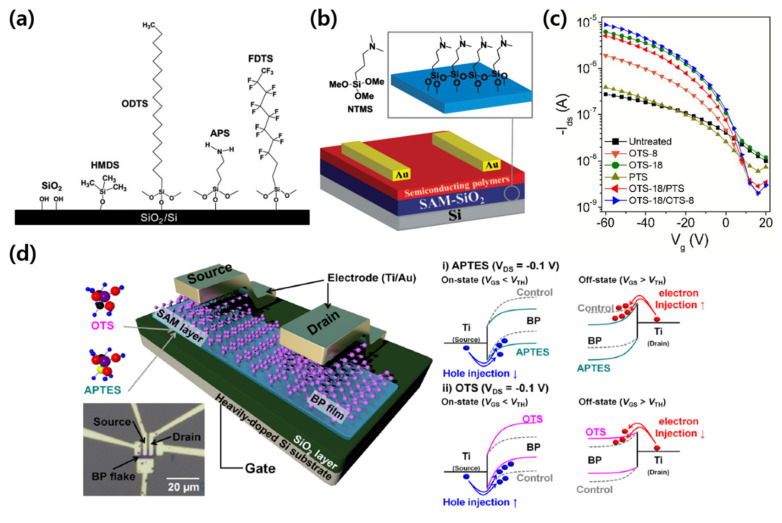
SAM treatment on SiO_2_ gate dielectrics. (**a**) Various SAMs on the SiO_2_ surface (adapted from [37] with permission from the Royal Society of Chemistry). (**b**) Structure of a pSNT transistor with NTMS-treated SiO_2_ and the chemical structure of NTMS (adapted from [38] with permission from John Wiley and Sons). (**c**) Transfer characteristics of a poly(3-hexylthiophene) (P3HT) transistor before/after various silane-based SAM treatments on SiO_2_ (adapted from [39] with permission from the Royal Society of Chemistry). (**d**) BP transistor with ODTS- and APTES-treated SiO_2_ gate dielectric and the energy band diagram before/after each SAM treatment (adapted from [40] with permission from the American Chemical Society).

Ku et al. fabricated a graphene field effect transistor (FET), in which several functional group SAMs were applied as buffer layers, and analyzed the effect of each SAM (Figure 1a) [37]. Among them, the graphene FET on 1H,1H,2H,2H-perfluorodecyltriethoxysilane (FDTS)-treated SiO_2_ showed a p-doping effect, whereas for the (3-aminopropyl) triethoxysilane (APTES)-treated SiO_2_, an n-doping effect was observed. These results suggest that it is important to control the influence of the SAM dipole in the fabrication of graphene FETs. Wang et al. reported that the performance of an n-type polymer pSNT transistor was improved by treating the SiO_2_ surface with [3-(N,N-dimethylamino) propyl] trimethoxysilane (NTMS), an amine-tailed SAM (Figure 1b) [38]. The SiO_2_ surface treatment improved the low on/off current ratio characteristic due to the hole current in the off state by a factor of 10. Lei et al. studied the optimal surface treatment for SiO_2_ used in OFETs by using octyltrichlorosilane (OTS), octadecyltrichlorosilane (ODTS), and phenyltrichlorosilane (PTS) as treatments (Figure 1c) [39]. When using the ODTS treatment, high mobility characteristics appeared, but a low on/off current ratio was observed owing to the high off current. In contrast, when using the OTS and PTS treatments, high on/off current ratio characteristics but low mobility characteristics were obtained. Therefore, a combination of ODTS, with high mobility characteristics, and OTS or PTS, with a high on/off current ratio, was applied. As a result, both high mobility characteristics and high on/off current ratio characteristics were achieved. Kang et al. reported the p-type and n-type doping of black phosphorus (BP) films on SiO_2_ substrates treated with ODTS and APTES, respectively (Figure 1d) [40]. They suggested that this is because the energy band structure of BP was changed by the dipole of the APTES and ODTS functional groups. In addition, the APTES and ODTS functional groups affected the optical characteristics. For the BP transistor on the APTES-treated SiO_2_, the photocurrent generated under light irradiation was reduced compared to that on the untreated SiO_2_. In contrast, for the BP transistor on the ODTS-treated SiO_2_, the photocurrent increased.

### 2.2. SAM Treatment on Al_2_O_3_ Gate Dielectrics

**Figure 2:**
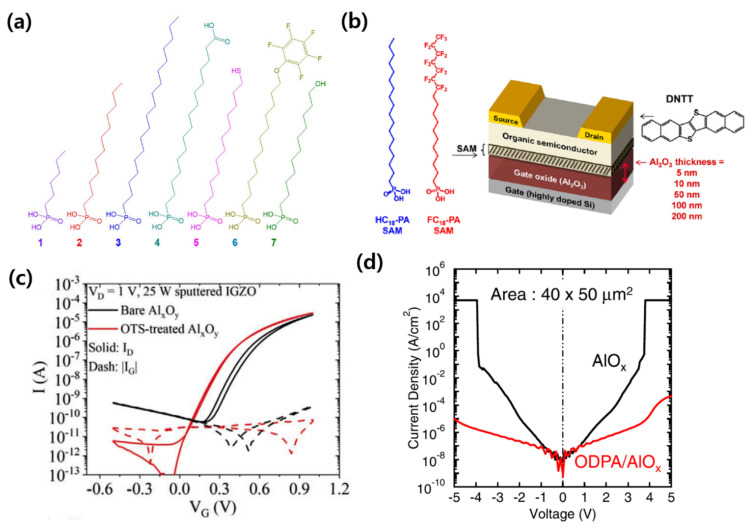
SAM treatment on Al_2_O_3_ gate dielectrics. (**a**) Chemical structures of various phosphonic acid SAMs. [1: Hexylphosphonic acid (HPA), 2: dodecylphosphonic acid (DDPA), 3: octadecylphosphonic acid (ODPA), 4: 16-phosphonohexadecanoic acid (PHDA), 5: 12-mercaptododecylphosphonic acid (MDPA), 6: 12-pentafluorophenoxydodecylphosphonic acid (PFPA), 7: 11-hydroxyundecylphosphonic acid (HUPA)] (adapted from [41] with permission from Springer Nature). (**b**) Schematic of a DNTT transistor to compare the effect of two phosphonic acid SAM treatments for the thickness of Al_2_O_3_ gate dielectrics (ODPA, FDPA) (adapted from [42] with permission from the American Chemical Society). (**c**) Transfer characteristics of an IGZO transistor with and without ODTS on Al_2_O_3_ (adapted from [43] with permission from John Wiley and Sons). (**d**) Comparison of the gate leakage current with or without the ODPA treatment of Al_2_O_3_ (adapted from [44] with permission from AIP Publishing).

In addition to the SAM treatment on SiO_2_, many advances of SAM-treated Al_2_O_3_ have been reported. Jang et al. treated the hydrophilic surface of Al_2_O_3_ with various phosphonic acid SAMs and compared the characteristics based on the alkyl chain length and functional end groups of the SAMs (Figure 2a) [41]. The longer the alkyl chain length, the more hydrophobic the surface, resulting in greater contact angle characteristics. The characteristics of phosphonic acid SAM according to functional end-groups were identified as phosphonohexadecanoic acid (PHDA), 12-mercaptododecylphosphonic acid (MDPA), 12-pentafluorophenoxydodecylphosphonic acid (PFPA), and 11-hydroxyundecylphosphonic acid (HUPA). Among these phosphonic acid SAMs, only PFPA, which has a non-polar functional end-group, exhibited hydrophobic properties similar to alkyl phosphate SAMs. It was established that PHDA, MDPA, and HUPA have high surface energy characteristics because their functional end-groups are polar. Aghamohammadi et al. investigated the relationship between the gate dielectric thickness and the threshold voltage of n-octadecylphosphonic acid (ODPA)- and 12,12,13,13,14,14,15,15,16,16,17,17,18,18,18-pentadecylfluoro-octadecylphosphonic acid (FDPA)-treated Al_2_O_3_ in DNTT transistors (Figure 2b) [42]. In the case of FDPA, when the Al_2_O_3_ thickness increased, the threshold voltage showed an inverse relationship with the dielectric capacitance. In contrast, for the ODPA-treated Al_2_O_3_, the threshold voltage was determined only by the SAM, regardless of the thickness of the Al_2_O_3_. Therefore, they suggested that the shift in threshold voltage for the SAM-treated gate dielectric was related not only to the dipole of the SAM material, but also to the interface between the SAM material and the semiconductor layer. Kawanago et al. analyzed the changes when ODTS-treated Al_2_O_3_ was applied as the gate dielectric of an oxide semiconductor-based transistor (Figure 2c) [43]. After the ODTS treatment, as the density of the trap between the channel and the gate dielectric decreased, the carrier mobility and on/off current ratio increased. Cai et al. used an ODPA-treated Al_2_O_3_ gate for the low-voltage operation of MoS_2_ [44]. The gate leakage current was significantly reduced by the ODPA treatment (Figure 2d).

### 2.3. SAM Treatment on HfO_2_ Gate Dielectrics

**Figure 3:**
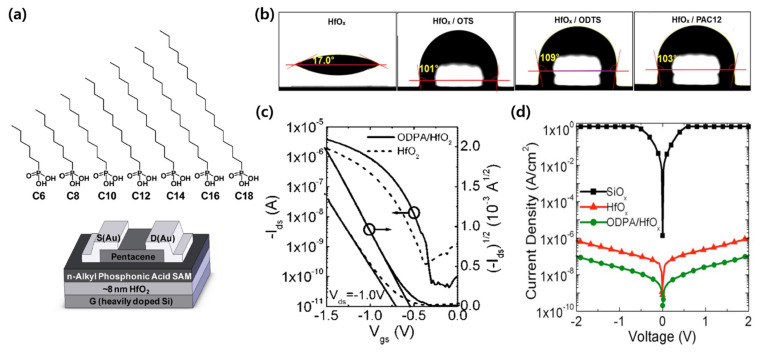
SAM treatment on HfO_2_ gate dielectrics. (**a**) Chemical structure of phosphonic acid SAMs with various carbon chain lengths; n-hexylphosphonic acid (HPA), n-octylphosphonic acid (OPA), n-decylphosphonic acid (DPA), n-dodecylphosphonic acid (DDPA), n-tetradecylphosphonic acid (TDPA), n-hexadecylphosphonic acid (HDPA), and n-octadecylphosphonic acid (ODPA) (adapted from [45] with permission from the American Chemical Society). (**b**) Water contact angle of pristine HfO_x_ and HfO_x_ treated with OTS, ODTS, and DDPA, respectively (adapted from [46] with permission from Elsevier). (**c**) Comparison of transfer characteristics with or without the ODPA treatment of HfO_2_ (adapted from [47] with permission from the American Chemical Society). (**d**) Comparison of the leakage current for SiO_x_, bare HfO_x_, and ODPA-treated HfO_x_ (adapted from [48] with permission from AIP Publishing).

As another high-k dielectric layer, HfO_2_ dielectric was applied by SAM treatments. Acton et al. showed that the performance of a low-voltage OTFT can be controlled by adjusting the carbon chain length of the alkyl PA SAM on the HfO_2_ insulating layer (Figure 3a) [45]. It was suggested that the high carrier mobility was determined by the balance between the SAMs that exhibited sufficient thickness and the disordered SAMs to prevent adverse effects in the high-k gate dielectric. Kim et al. introduced hydrophobic properties on the hydrophilic HfO_2_ surface by incorporating OH groups through OTS, ODTS, and n-dodecylphosphonic acid (DDPA) (Figure 3b) [46]. With this, they produced a poly-(2,5-bis(2-decyltetradecyl)-3-(3″,4′-difluoro-[2,2′:5′,2″-terthiophen]-5-yl)-6-(thiophen-2-yl) pyrrolo [3,4-c] pyrrole-1,4-(2H,5H)-dione) (PDPP2DT-T2)-based organic transistor capable of low voltage operation (<−4 V) with reduced trap density. Ting et al. studied the effects of SAMs containing different binding groups, such as ODPA, 4,5-dioctadecyl-benzene1,2-diol (C36C), 4-Octadecyl-benzene-1,2-diol (C18C), and stearic acid (SA) [47]. Among the four SAMs, the ODPA treatment resulted in a low leakage current density and the largest contact angle with water. In addition, dialkylcatechol showed similar properties to ODPA, indicating that a catechol-based SAM is suitable for HfO_2_ surface treatment. Finally, they demonstrated a pentacene TFT capable of operating under −1.5 V with an improved on/off current ratio through HfO_2_ surface treatment (Figure 3c). Acton et al. fabricated a poly (2,5-bis(3-tetradecyl-5-(3-tetradecylthiophen-2-yl)thiophen-2-yl) thiazolo [5,4-d] thiazole) (PTzQT-14) TFT that is flexible and capable of low voltage operation using an ODPA-treated HfO_2_ insulating film [48]. The ODPA treatment reduced the gate leakage current (Figure 3d), and, above all, the flexible TFT can possibly be applied as a flexible and wearable low-voltage device capable of low voltage operation owing to the extreme thinness of the SAM material.

### 2.4. SAM as Gate Dielectrics

**Figure 4:**
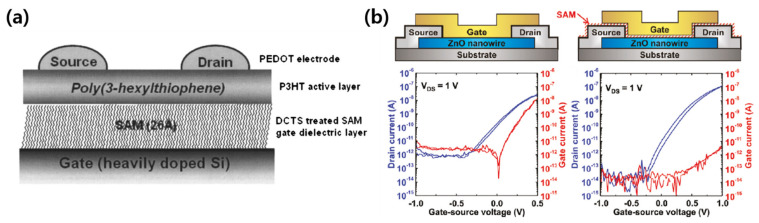
SAMs as gate dielectrics. (**a**) Schematic of P3HT transistor using docosyltrichlorosilane (DCTS) as a gate dielectric (adapted from [50] with permission from AIP Publishing). (**b**) Comparison of the electrical characteristics in MISFET and MESFET structures of ZnO nanowire FET with or without a SAM gate dielectric (adapted from [51] with permission from the American Chemical Society).

Since several SAMs themselves have good insulating properties, a low-voltage operation of the transistor by the SAM gate dielectric without an oxide dielectric was achieved. Halik et al. applied (18-phenoxyoctadecyl)trichlorosilane (PhO-OTS), a silane-based SAM material, as the gate dielectric of a pentacene TFT [49]. They applied the SAM gate dielectric in both a bottom-gate bottom-contact structure and a bottom-gate top-contact structure. In addition, the SAM gate dielectric, which is 2.5 nm thick, makes it possible to operate at voltages of less than 2 V. They also suggested that the SAM gate dielectric is capable of low-voltage and low-power operation, even in organic devices. A solution-processed poly(3-hexylthiophene) (P3HT) polymer transistor using a docosyltrichlorosilane (DCTS) SAM dielectric was demonstrated (Figure 4a) [50]. Kälblein et al. prepared a top-gate structure ZnO nanowire transistor in which ODPA, a phosphonic acid-based SAM material, was applied as the gate dielectric [51]. To confirm whether ODPA is effective as a gate dielectric, the electrical characteristics of the metal-semiconductor FET (MESFET) structure without the ODPA gate dielectric and the metal-insulator-semiconductor FET (MISFET) structure with the ODPA gate dielectric were compared (Figure 4b). The electrical characteristics of the MESFET structure without the ODPA gate dielectric and the MISFET structure with the ODPA gate dielectric were compared. The MESFET structure without the ODPA gate dielectric exhibited a high gate leakage current flow. However, the presence of the ODPA gate dielectric greatly reduced the gate leakage current, which improved the on/off current ratio. Therefore, the dielectric properties of ODPA were confirmed, and because of the thin thickness of the SAM, it was possible to operate the ZnO nanowire transistor at 1 V.

### 2.5. Self-Assembled Monolayer Field Effect Transistor (SAMFET)

**Figure 5:**
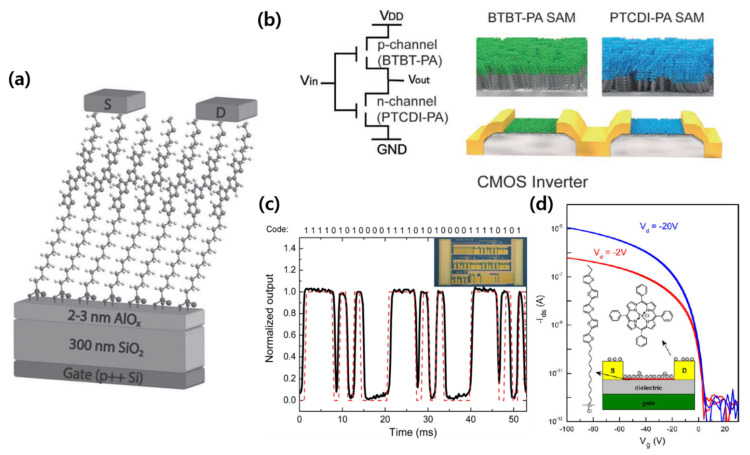
SAMs as semiconductor layers. (**a**) Schematic of a SAMFET based on phosphonic acid SAM (adapted from [52] with permission from John Wiley and Sons). (**b**) Schematic and illustration of a SAMFET-based CMOS inverter (adapted from [53] with permission from John Wiley and Sons). (**c**) The output of a 4-bit code generator incorporating over 100 SAMFETs. The red dotted line indicates the preprogrammed code (adapted from [54] with permission from the American Chemical Society). (**d**) Typical SAMFET transfer characteristic and the schematics of a SAM-based gas sensor (adapted from [55] with permission from Elsevier).

A self-aligned structure of SAMs enabled their operation as a semiconductor active layer through the π–π overlap in the molecular packing. Cernetic et al. fabricated a p-type SAMFET with (11-(5‴-(4-(methylthio)butyl)-[2,2′:5′,2″:5″,2‴-quaterthiophene]-5-yl)undecyl)phosphonic acid (MTB4TC11) and (12-(5‴-(4-(methylthio)butyl)-[2,2′:5′,2″:5″,2‴-quaterthiophene]-5-yl) dodecyl) phosphonic acid (MTB4TC12), which are phosphonic acid-based SAMs [52]. Figure 5a shows the schematic of the fabricated SAMFET. They improved the contact characteristics with the Au electrode by applying methylthiobutyl as a functional terminal group. In addition, the bonding between the SAM and the electrode was strengthened through annealing, so that the carrier mobility could be increased by more than 100-fold. By applying these optimized conditions, a SAMFET capable of low-voltage operation through a HfO_2_ gate dielectric was produced. Zhao et al. implemented two organic inverters using a SAMFET [53]. First, after fabricating an ambipolar transistor with a semiconducting SAM and a complementary semiconductor, they established a CMOS-like inverter with two anti-ambipolar transistors. Second, a CMOS inverter was produced by fabricating a benzothieno [3,2-b] [1] benzothiophene (BTBT)-PA-based p-type SAMFET and a 3,4,9,10-perylene tetracarboxylic diimide (PTCDI)-PA-based n-type SAMFET (Figure 5b). They presented the possibility of developing organic circuits by producing the aforementioned SAM-based organic inverters over a large area for the first time. Gholamrezaie et al. suggested that semiconducting SAMs can be grown on organic dielectrics and that charge transport is possible even at micrometer distances [54]. They also reported the use of a 4-bit code generator incorporating over 100 SAMFETs (Figure 5c). Andringa et al. reported the application of Fe(TPP)Cl as an NO receptor to a SAMFET and used it as a gas sensor (Figure 5d) [55]. They suggested that a very thin SAMFET channel, approximately one molecule thick, is suitable for use as a sensor.

## 3. SAMs as Dopants

In the second section, the effects of SAMs acting as dopants on the device’s properties are introduced. In the last decade, promising semiconductor materials, including transition metal dichalcogenides (TMDs) [56,57,58,59,60], oxides [61,62,63,64,65], and polymers [66,67,68,69,70], have emerged as next-generation semiconductors. However, the conventional doping techniques (i.e., ion implantation) used in silicon-based fabrications degrade and damage these semiconductors; thus, there is a need for the development of alternative methods to control the electrical properties of the semiconductors. To meet these demands, unusual SAM-based doping techniques have been attempted [71,72,73,74,75,76,77,78,79,80]. Owing to the ordered domain and large area coverage of SAMs, uniform and controllable doping was achieved. An expansive overview of SAM-based doping into oxides and TMDs is presented in this section.

### 3.1. Doping Effects of SAMs in Oxide Semiconductors

**Figure 6:**
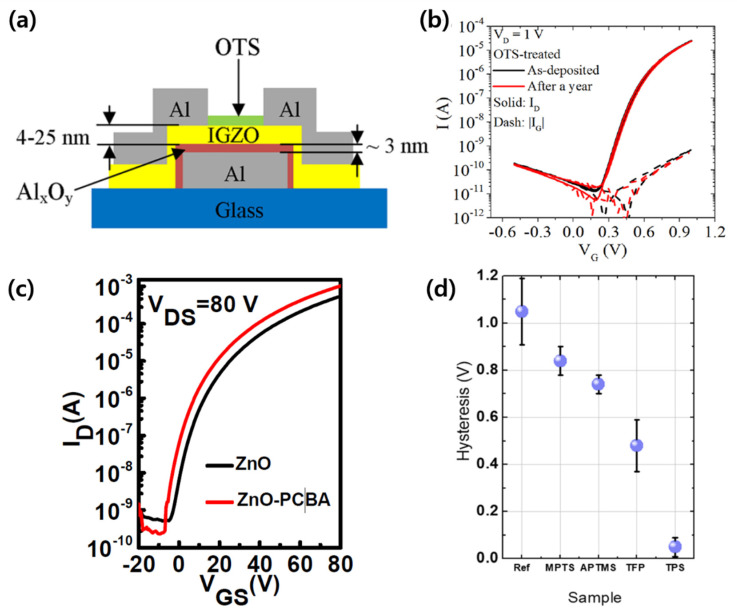
SAMs as dopants in oxide semiconductor-based transistors (**a**) Schematic of ODTS-treated IGZO TFT (adapted from [79] with permission from the American Chemical Society). (**b**) Transfer characteristics of IGZO TFT immediately after ODTS treatment and 1 year later (adapted from [79] with permission from the American Chemical Society). (**c**) Comparison of pristine ZnO TFT and PCBA-treated ZnO TFT transfer characteristics (adapted from [81] with permission from MDPI). (**d**) Hysteresis characteristics of IGZO TFT according to SAM (adapted from [78] with permission from the American Chemical Society).

In recent years, the use of SAMs as a layer of remote doping material for prefabricated transistors, not as an insulating film or a semiconductor, has been attempted. As an example of this doping technique of an SAM on a metal oxide semiconductor, Cai et al. treated an IGZO surface with ODTS to form a passivation layer (Figure 6a), which greatly increased the carrier mobility [79]. In addition, without the passivation layer, the device significantly deteriorated after 1 year, whereas the device containing the passivation layer maintained its performance after 1 year (Figure 6b). This indicates that the passivation layer introduced through the SAM treatment protected the device from oxygen derivatives from the air over an extended period. Wan et al. improved electron mobility and the on/off current ratio through the 4-chlorobenzoic acid (PCBA) treatment of ZnO transistors (Figure 6c) [81]. They extracted the trap concentration from the electrical characteristics of a pristine ZnO transistor and a PCBA-treated ZnO transistor and found that it decreased by about 10-fold after PCBA treatment. Lee et al. studied the surface treatment of IGZO TFTs with SAMs [78]. The results showed that by selecting a SAM with an appropriate functional group polarity and alkyl chain length, the electron transport could be improved and the hysteresis reduced (Figure 6d). In addition, it was shown that the contact resistance can be controlled by the alkyl chain length when using the same functional group. A similar result was achieved by Xiao et al. for IGZO TFTs [75]. Xiao et al. analyzed the effect of passivating the surface of an IGZO TFT with triethoxysilane (TES)-based SAMs. In addition, three types of TESs containing different carbon chain lengths, namely methyltriethoxysilane (MTES), octyltriethoxysilane (OTES), and oxtadecyltriethoxysilane (ODTES), were used to determine the effect of the alkyl chain length on the semiconductor characteristics. As a result, the longer the alkyl chain length, the more improved all the electrical characteristics, such as carrier mobility and hysteresis.

### 3.2. Electrical Doping Effect of SAMs in 2D Materials

**Figure 7:**
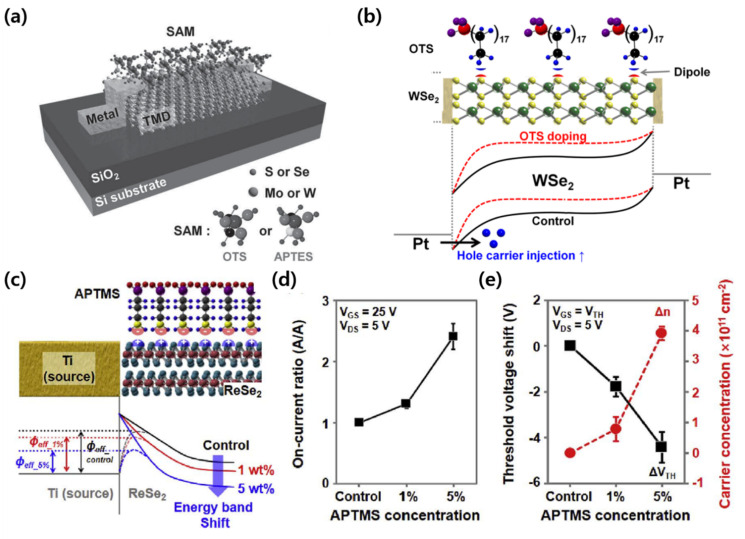
SAMs as dopants in TMD-based transistors. (**a**) Schematic of a SAM-doped TMD-based transistor (adapted from [72] with permission from John Wiley and Sons). (**b**) Energy band diagram of a WSe_2_ transistor before/after ODTS doping (adapted from [73] with permission from the American Chemical Society). (**c**) Charge polarity at the APTMS-ReSe_2_ interface and the change of energy band diagram after the doping of an ReSe_2_ transistor by APTMS concentration (1 and 5 wt%). Comparison of (**d**) on-current ratio, (**e**) threshold voltage shift, and carrier concentration according to APTMS concentration (adapted from [74] with permission from Elsevier).

The SAM doping techniques were attempted in 2D TMDs. Due to the atomically thin nature of the TMDs, the SAM doping was more effective in improving device performance. Kang et al. reported improved electrical properties for MoS_2_ and WSe_2_ transistors by doping with ODTS and APTES SAM materials (Figure 7a) [72]. When the MoS_2_ transistors were doped with APTES and the WSe_2_ transistors with ODTS, the field-effect mobility increased by approximately fivefold. In contrast, when doping the MoS_2_ transistor with ODTS and the WSe_2_ transistor with APTES, the field-effect mobility deteriorated. As ODTS and APTES exhibited p-doped and n-doped effects, the n-type MoS_2_ could be improved by APTES doping, and the p-type WSe_2_ could be improved by ODTS doping. They also suggested that the p-doping effect of the ODTS-doped WSe_2_ transistor was owing to the functional group of the ODTS [73]. Due to the positive charges of the methyl functional groups (-CH_3_) in ODTS, the electrons in WSe_2_ are attracted to the junction with ODTS, which leads to a decrease in the concentration of electrons in the WSe_2_ channel and a p-doping effect (Figure 7b). Hasnain et al. studied the doping effect of (3-aminopropyl)trimethoxysilane (APTMS) in ReSe_2_ transistors with n-type characteristics as a function of the APTMS concentration (Figure 7c–e) [74]. They suggested that the electron concentration in the ReSe_2_ channel increased because of the negative charges of the amine functional groups (-NH_2_) of APTMS at the interface between APTMS and ReSe_2_. As a result, the n-doping effect of APTMS reduced the effective barrier height between ReSe_2_ and the electrode, and the threshold voltage shifted in the negative direction. After doping with APTMS, these effects increased the photoresponsivity for all wavelength bands, not only in terms of electrical characteristics, but also in terms of the photoresponsive properties.

## 4. Biosensor Linkers Based on SAMs

Finally, we revisit the recent advances and studies in biosensors that use SAM-based linkers. Recent viral diseases and pandemics have generated interest in various types of sensors, such as electrochemical impedance spectroscopy (EIS) [82,83,84] and field-effect biosensors (BioFETs) [85,86,87,88,89]. These devices can provide warnings on a patient’s dangerous condition or offer daily monitoring information. Biosensors should exhibit crucial functions, including fast detection and response [90], robust operation [91], and the detection of low-concentration targets [92], for reliable detection systems. To realize these functions, a linker with an ordered domain should be located between the active layer and the analytes to anchor the analytes (i.e., target antibodies and enzymes). As molecular assemblies allow SAMs to be tightly packed and oriented on the active layer, a dense domain of analytes can be formed. In this section, we provide an overview of biosensors for antigen and biomarker detection enhanced by using SAM-based linkers.

**Figure 8:**
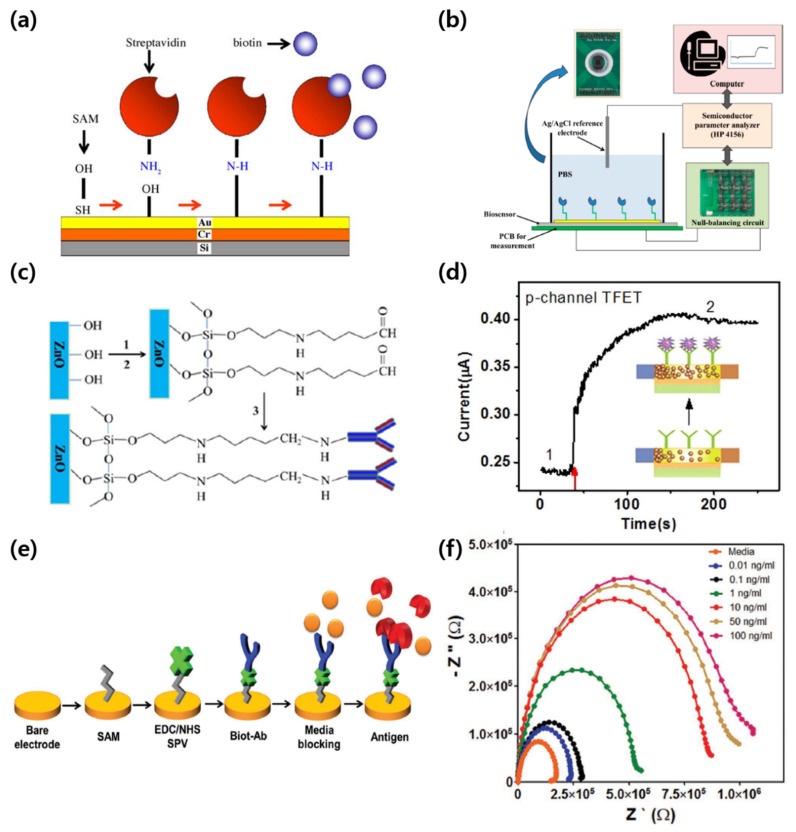
SAMs as a linker in biosensors. (**a**) Schematic of the immobilization sequence of streptavidin and the interaction between streptavidin and biotin (adapted from [93] with permission from Elsevier). (**b**) Measurement setup to detect C-reactive protein (adapted from [94] with permission from MDPI). (**c**) Immobilization of uricase onto the ZnO NW surface via the crosslinking surface modification method: (1) 2% APTES in ethanol; (2) 25 wt% GAD; (3) 5μL uricase (5 units/mL) (adapted from [88] with permission from Elsevier). (**d**) Electrical characteristics of an SiNW-TFET based biosensor detecting CYFRA21-1 (adapted from [85] with permission from Springer Nature). (**e**) Illustration of immobilizing antibodies onto the microelectrodes and reacting with antigens (adapted from [95] with permission from John Wiley and Sons). (**f**) Nyquist plots for different standard human albumin concentrations. Media refers to the treatment of cell culture media to prevent the nonspecific binding of proteins (adapted from [95] with permission from John Wiley and Sons).

Kim et al. studied an extended gate FET (EGFET)-based biosensor for detecting streptavidin–biotin protein complexes [93]. The sequence of immobilization of streptavidin to the gate electrode is shown in Figure 8a. They used a C11-oligo(ethylene glycol)-terminated (OEG) thiol SAM with thiol and hydroxyl groups to immobilize streptavidin (SPV) on the Au gate electrode. The OEG thiol SAM head group thiols, was bonded to the Au gate electrode to form a hydroxyl group on the surface. Then, streptavidin was immobilized by combining the amine group of streptavidin with the surface’s hydroxyl group. Similarly, Lee et al. demonstrated a biosensor based on a high electron mobility transistor (HEMT) for C-reactive protein (CRP) detection by immobilizing the antibody on the gate [94]. To immobilize the receptor, CRP, a carboxyl group was first formed on the gate surface with 11-mercaptoundecanoic acid(11-MUA), and then a stable amine-reactive product was formed with N-(3-dimethylaminopropyl)-N′-ethylcarbodiimide hydrochloride (EDC) and N-hydroxysuccinimide (NHS). Then, CRP-antibody immobilization was completed. Figure 8b shows the AlGaN/GaN HEMT-based biosensor configuration for CRP detection. They detected CRP by measuring not only the change in drain current but also the change in the output voltage using a null-balancing circuit. In addition to immobilizing the receptor on the gate electrode, a method of immobilizing the receptor on the channel layer has also been reported. Liu et al. immobilized uricase on a ZnO nanowire channel to fabricate a sensor that detects uric acid [88]. Figure 8c shows the process of immobilizing uricase. First, the hydroxyl group on the ZnO nanowire surface was converted into an amine group by silane-based APTES treatment. Then, the surface modification was completed by linking the uricase and the amine group through glutaraldehyde. Gao et al. demonstrated a tunnel field-effect transistor (TFET)-based biosensor capable of detecting CYFRA21-1, a lung cancer biomarker [85]. They first formed amine groups on the Si nanowire channel by APTES treatment. Subsequently, the amine group on the Si nanowire surface was reacted with glutaraldehyde, and then the CYFRA21-1 antibody was immobilized. When the CYFRA21-1 antigen and antibody were combined, the electrical properties acting on the channel region was changed, influenced by the polarity of the biomolecule. The energy band structure of the TFET and the tunneling current were also modulated (Figure 8d). As a result, the presence of CYFRA21-1 antigen could be detected through the change of the current of the TFET. Shin et al. demonstrated a biosensor for detecting biomarkers related to liver health based on EIS [95]. Starting with the SAM treatment, the antibody’s binding process to the Au electrode is shown in Figure 8e. The Au electrode was treated with 11-MUA SAM, and NHS ester was formed through EDC/NHS. Then, SPV was immobilized by reacting the surface with the amine group of SPV. Finally, it was completed by immobilizing biotinylated antibodies (biot-Abs) to SPV. The detection of the target in the fabricated sensor was achieved through the change in impedance as shown in Figure 8f.

## 5. Summary

We first revisited examples of using SAM for the surface treatment of oxide gate dielectrics in electronics. Oxide dielectrics had problems due to charge traps caused by hydroxyl groups on the surface and problems with leakage current flowing in high-k dielectrics such as Al_2_O_3_ and HfO_2_. These problems in the oxide dielectric were addressed by the SAM; the SAMs reacting with the hydroxyl groups on the dielectric surface were treated. The increased hydrophobicity can improve device performance because it minimizes the absorption of oxygen or water molecules that result in poor device stability and performance degradation. As the SAM treatment reduced the charge trap by allowing the hydrophilic surface to become hydrophobic. The decrease in trap density led to improved electrical characteristics such as high on/off current ratio and the enhanced mobility of the devices. Besides, several researchers have experimentally proven that there is also an effect by the carbon chain of SAM. This is because the longer the carbon chain length is, the better the SAM molecules can be ordered and the more hydrophobic the surface is.

Interestingly, SAMs themselves are also used as gate dielectrics or semiconductor layers. Table 1 shows examples of applying SAMs to the gate dielectric surface treatment and examples of using a SAM itself as a gate dielectric. The possibility of applying a molecularly thin SAM as a gate dielectric mean that the device can be operated with low voltage. Based on these research results, this has been demonstrated for SAM gate dielectric- and SAMFET-based functional circuit-levels such as NANDs, ring oscillators, and code generators. This shows that SAMs have great potential in electronics.

The utility of SAMs is not limited to basic elements of devices such as gate dielectrics and semiconductors, but is also used as a dopant for devices. TMDs and oxide semiconductors, emerging as next-generation semiconductor materials, have difficulty applying the ion implantation process for doping conventional silicon semiconductors. As an alternative to the conventional doping method, which is difficult to apply, studies have been conducted to provide a doping effect by placing a SAM on a fabricated device. We have summarized examples of doping attempts using SAM in Table 2. The doping effect in oxide semiconductors and TMDs is slightly different. In oxide semiconductors, the hydroxyl group acting as a trap on the surface of the semiconductor layer decreases through reaction with the head group of the SAM, thereby eliminating the trapping sites and increasing the number of carriers in the channel, thereby improving the device performance [78]. On the other hand, in TMDs, the characteristics of the TMD-based transistor are modulated due to the effect of the tail group of the SAM facing the semiconductor layer, and the dipole of the tail group applies an electric field to the channel [71].

Finally, we dealt with the use of SAMs as a linker in biosensors. In biosensors, it is necessary to have selectivity for the specific target to be detected, such as an antigen, and the ability to detect if even a small concentration of the target is present is important. Therefore, it is necessary to allow the maximum interaction between the target and the receptor to occur. As a method, there is alignment by immobilizing receptors such as antibodies in a specific direction. However, since not all receptors can be directly immobilized on various substrates, SAMs are widely applied to connect and immobilize the substrate and the receptor as a linker.

As there are many advantages, shown through various examples, of using SAMs, further advances in electronics will be in progress. SAM-based electronic devices have the following merits and directions for future research: (1) low-cost, (2) device flexibility, (3) work-function modification, and (4) device stability enhancement. Owing to the solution processability of SAMs, the SAM-based device process can be performed through simple spin-coating or printing, which reduces the fabrication process complexity, and thus lowers the cost. Furthermore, the low thermal budget of the SAM-based process enhances the compatibility with flexible substrates (i.e., polyimide and polyethylene terephthalate). These merits provide great ease in implementing flexible devices such as communication devices and sensors [96,97]. In addition to the fabrication processing aspect, SAMs enable the contribution towards device aspects. The work function modification can be achieved by SAM surface treatment due to its molecularly thin thickness and assembly reaction. This property ensures that the work function of the electrodes matches what can provide the desired energy band junction for each device [98,99]. The SAMs also improve the device stability. The SAM can control surface properties into hydrophobicity, minimizing the absorption of oxygen or water molecules that act as charge trap sites. There are still difficulties such as the improvement of the integration process, miniaturization, and pattern refinement in SAM-based electronics. However, the advantages and possibilities of the aforementioned SAMs are expected to lead to the next generation of electronic devices.

## Figures and Tables

**Table 1:** Various types of self-assembled monolayers (SAMs) for gate dielectrics.

Name	Type	Head Group	End Group	Method	Bottom Layer	Operating Voltage	Ref.
NTMS	Silane	Trimethoxysilane (-Si(OCH_3_)_3_)	Amine (-NH_2_)	Spin-coating	SiO_2_ (285 nm)	80 V	[38]
APTES	Silane	Triethoxysilane (-Si(OC_2_H_5_)_3_)	Amine (-NH_2_)	Dipping	SiO_2_ (285 nm)	−40 V	[40]
OTS	Silane	Trichlorosilane (-SiCl_3_)	Methyl (-CH_3_)	Dipping	SiO_2_ (285 nm)	−40 V	[40]
PTS	Silane	Trichlorosilane (-SiCl_3_)	Phenyl (-C_6_H_5_)	N/A	SiO_2_ (N/A)	−60 V	[39]
MTS	Silane	Trichlorosilane (-SiCl_3_)	Methyl (-CH_3_)	N/A	SiO_2_ (N/A)	−60 V	[39]
ODTS	Silane	Trichlorosilane (-SiCl_3_)	Methyl (-CH_3_)	Dipping	SiO_2_ (300 nm)	−80 V	[37]
FDTS	Silane	Triethoxysilane (-Si(OC_2_H_5_)_3_)	Trifluoromethyl (-CF_3_)	Dipping	SiO_2_ (300 nm)	−80 V	[37]
HPA	Phosphonic acid	Phosphonic (-PO(OH)_2_)	Methyl (-CH_3_)	Spin-coating	Al_2_O_3_ (N/A)	−4 V	[41]
DDPA	Phosphonic acid	Phosphonic (-PO(OH)_2_)	Methyl (-CH_3_)	Spin-coating	Al_2_O_3_ (N/A)	−4 V	[41]
PHDA	Phosphonic acid	Phosphonic (-PO(OH)_2_)	Carboxyl (-COOH)	Spin-coating	Al_2_O_3_ (N/A)	−4 V	[41]
MDPA	Phosphonic acid	Phosphonic (-PO(OH)_2_)	Thiol (-SH)	Spin-coating	Al_2_O_3_ (N/A)	−4 V	[41]
PFPA	Phosphonic acid	Phosphonic (-PO(OH)_2_)	Pentafluorophenoxy	Spin-coating	Al_2_O_3_ (N/A)	−4 V	[41]
HUPA	Phosphonic acid	Phosphonic (-PO(OH)_2_)	Hydroxyl (-OH)	Spin-coating	Al_2_O_3_ (N/A)	−4 V	[41]
FDPA	Phosphonic acid	Phosphonic (-PO(OH)_2_)	Trifluoromethyl (-CF_3_)	Dipping	Al_2_O_3_ (5 nm)	−2.5 V	[42]
ODPA	Phosphonic acid	Phosphonic (-PO(OH)_2_)	Methyl (-CH_3_)	Dipping	Al_2_O_3_ (5 nm)	−2.5 V	[42]
ODPA	Phosphonic acid	Phosphonic (-PO(OH)_2_)	Methyl (-CH_3_)	Dipping	X	1.5 V	[51]
PhO-OTS	Silane	Trichlorosilane (-SiCl_3_)	Phenyl (-C_6_H_5_)	Vapor-phase deposition	X	−2.1 V	[49]
DCTS	Silane	Trichlorosilane (-SiCl_3_)	Methyl (-CH_3_)	Dipping	X	-2 V	[50]

**Table 2:** Self-assembled monolayers (SAMs) used as a dopant.

Name	Type	Head Group	End Group	Method	Bottom Layer	Doping Type	Ref.
ODTS	Silane	Trimethoxysilane (-Si(OCH_3_)_3_)	Methyl (-CH_3_)	Spin-coating	Graphene	P-type	[71]
ODTS	Silane	Trichlorosilane (-SiCl_3_)	Methyl (-CH_3_)	Dipping	WSe_2_	P-type	[72,73]
ODTS	Silane	Trichlorosilane (-SiCl_3_)	Methyl (-CH_3_)	Spin-coating	IGZO	N-type	[79]
OTES	Silane	Triethoxysilane (-Si(OC_2_H_5_)_3_)	Methyl (-CH_3_)	Vapor-phase deposition	ITZO	N-type	[76,77]
APTES	Silane	Triethoxysilane (-Si(OC_2_H_5_)_3_)	Amine (-NH_2_)-	Dipping	MoS_2_	N-type	[72]
APTMS	Silane	Trimethoxysilane (-Si(OCH_3_)_3_)	Amine (-NH_2_)-	Dipping	RSe_2_	N-type	[74]
